# Vitamin D Deficiency in Children with a Chronic Illness–Seasonal and Age-Related Variations in Serum 25-hydroxy Vitamin D Concentrations

**DOI:** 10.1371/journal.pone.0060856

**Published:** 2013-04-09

**Authors:** Elisa Holmlund-Suila, Panu Koskivirta, Tuula Metso, Sture Andersson, Outi Mäkitie, Heli T. Viljakainen

**Affiliations:** 1 Children’s Hospital, Helsinki University Central Hospital and University of Helsinki, Helsinki, Finland; 2 Skin and Allergy Hospital, Division of Allergy, Helsinki University Central Hospital, Helsinki, Finland; 3 Musculoskeletal Research Unit, University of Bristol, Bristol, United Kingdom; 4 Folkhälsan Research Center, Helsinki, Finland; Oklahoma State University, United States of America

## Abstract

**Introduction:**

Children and adolescents with a chronic illness have potential risk factors for vitamin D deficiency. An optimal vitamin D status might have multiple health effects. This study evaluated vitamin D status and its association with age, gender, and season in a large cohort of chronically ill Finnish patients at a tertiary pediatric outpatient clinic. A cross-sectional register-based study was carried out, involving altogether 1351 children (51% boys, age range 0.2–18 years), who visited the outpatient clinic during 2007–2010 and had their vitamin D status (S-25-OHD) determined. A post-doc analysis was conducted to identify predisposing and preventing factors for vitamin D deficiency.

**Results:**

Almost half (47%) of the S-25-OHD values were consistent with subnormal vitamin D status (S-25-OHD <50 nmol/L) while only 12% were >80 nmol/L. Age and season were the most important determinants for S-25-OHD concentration. Mean S-25-OHD concentration differed between age groups (Kruskal-Wallis; p<0.001), adolescents being at highest risk for vitamin D insufficiency. Young age and vitamin D supplementation were preventive factors for deficiency, while non-Finnish ethnic background was a predisposing factor. S-25-OHD showed significant seasonal variation in children older than 6 years. In the whole cohort, S-25-OHD was on average 13 nmol/L higher in summer than in winter, and the prevalence of vitamin D deficiency ( =  S-25-OHD <37.5 nmol/l) varied from 11% in summer to 29% in winter.

**Conclusions:**

The finding that almost half of the studied Finnish children with a chronic illness had suboptimal vitamin D status is alarming. Inferior vitamin D status was noted in adolescents compared with younger children, suggesting that imbalance between intake and requirement evolves with age. Although less common during summer, subnormal vitamin D status was still observed in 28% of those evaluated in summer. Clinicians should identify individuals at risk and actively recommend vitamin D supplementation.

## Introduction

Vitamin D is a key nutrient related to well-being and growth especially in pediatric population [1]. Today nearly 40 tissues are characterized as target organs for vitamin D [2]; thus its effects expand far beyond skeletal homeostasis. Vitamin D deficiency in infancy increases the risk of upper respiratory tract infections [3] and poor growth [4], and long-term consequences of vitamin D deficiency are associated to the development of several chronic diseases [5], [6].

Serum 25-OHD is a reliable marker of vitamin D status. It combines sources of vitamin D: diet and solar exposure. Highest S-25-OHD concentrations are noted in farmers and lifeguards with constantly high UVB exposure [7] while lowest values are observed in northern latitudes where scarce sunlight is often accompanied with limited dietary intake of vitamin D. Recently, three independent expert panels have reviewed the evidence on S-25-OHD for several outcomes. Lawson Wilkins Pediatric Endocrine Society concluded that concentrations below 37.5 nmol/l are suggestive of vitamin D deficiency and concentrations between 37.5 and 50 nmol/L, suggestive of vitamin D insufficiency in children [8]. Institute of Medicine (2011) added that concentrations over 50 nmol/l are required for normal function of body including linear growth and bone mass accrual [9], while for optimizing long-term health such as prevention of diabetes or fractures concentrations above 75 nmol/l may be needed according to Endocrine Society [10].

Hypovitaminosis D is prevalent in Finnish children [11], [12]. In our recent school-based cohort approximately 70% of Finnish children had S-25-OHD below 50 nmol/L [13]. Compared with apparently healthy subjects, children with chronic illness may have additional risk factors for vitamin D deficiency; these may be related to the underlying chronic illness, its treatment or related factors (e.g. inflammation). However, children with chronic illness are usually under careful pediatric follow-up and more likely to have proper vitamin D supplementation. Current study was carried out firstly to evaluate vitamin D status and its association with gender, age and season in a large cohort of chronically ill Finnish children. Secondly, we wanted to define factors that predispose to or protect from vitamin D deficiency in these children.

## Subjects and Methods

### Study Cohort

This study is a register-based cross-sectional study on 1351 children, who visited the pediatric outpatient clinics at the Childreńs Hospital, Helsinki University Central Hospital, during 2007–2010 and had their vitamin D status (S-25-OHD) determined as part of routine follow-up. Helsinki is located in southern Finland (60○N) and the Childreńs Hospital is the largest pediatric hospital in the country. In the hospital region approximately 10% of inhabitants are of non-Finnish background and less than 5% are non-Caucasian [14]. Subjects included in this study had one or several chronic diseases, including asthma, allergies, gastrointestinal diseases, cancer, renal diseases, diabetes and other endocrine diseases, chronic inflammatory or infectious diseases, eating disorders or metabolic bone diseases, for which they required follow-up at a tertiary centre; hospital inpatients were not included. S-25-OHD measurements were obtained from the database of the Hospitaĺs Central Laboratory (HUSLAB, Hospital District of Helsinki and Uusimaa), where all the samples had been analysed. S-25-OHD measurements were based on the judgment of clinician in charge of patient care. Several patients had repeat measurements during follow-up but only the first measurement obtained during the study period 2007–2010 was included in the analyses. Baseline characteristics including age, gender, date of measurement and other laboratory analyses obtained during the same visit were available and collected from the database; no information regarding the patients’ ethnic background or the underlying clinical condition were available in the laboratory database.A post-hoc analysis was conducted to identify predisposing and preventing factors for vitamin D deficiency. From the initial population (N = 1351) we selected 100 subjects for post-hoc analysis: 50 children with the highest, and 50 with the lowest S-25-OHD values. The groups are called HIGH and LOW, respectively. Clinical characteristics including age, gender, ethnicity, key anthropometry, diagnosis and features of the underlying illness, date of vitamin D sampling and results of other blood work obtained during the same visit were collected from hospital records for each subject. The use of register-based data and the post-hoc analysis were approved by the Research Ethics Committee of the Hospital District of Helsinki and Uusimaa.

### Laboratory Measurements

S-25-OHD concentration (S-25-OHD total, S-25-OHD2 and S-25-OHD3) was analysed by high-performance liquid chromatography (HPLC) [15]. The detection limit is 10 nmol/L and the laboratory reference range >40 nmol/L for all age groups. Values below the detection limit (N = 4) were recorded as 5 nmol/L and included in the analysis. S-25-OHD concentrations were divided into four categories: <37.5 nmol/L, 37.5–50.0 nmol/L, 50.1–80.0 nmol/L and >80.0 nmol/L. S-25-OHD concentrations below 37.5 nmol/L were considered as indicative of vitamin D deficiency and 37.5–50.0 nmol/L as indicative of vitamin D insufficiency [8]. S-25-OHD concentrations between 50.1 to 80.0 nmol/L were considered to be sufficient [9] although some adult studies report beneficial skeletal effects when S-25-OHD concentration is above 80.0 nmol/L [10].

PTH was analysed with an immune chemiluminometric assay. The reference range for children and adults is 8–73 ng/L. Plasma creatinine (P-Cr) concentration was analysed by photometric enzymatic assay and reference values are compatible with the Nordic Reference Interval Project: 8 days-2 years 10–56 µmol/L, 3–5 years 10–48 µmol/L, 6–12 years 10–76 µmol/L, girls 13–16 years 15–90 µmol/L, boys 13–16 years 20–95 µmol/L, girls 17–18 years 40–90 µmol/L and boys 17–18 years 50–95 µmol/L.

### Clinical Data

Height (cm) and weight (kg) were collected from patient records for the 100 subjects in HIGH and LOW groups. Heights are reported in standard deviation (SD) units and weights as height-adjusted values (a percentage of the mean weight in gender- and height-adjusted reference population), according to the Finnish standards [16], [17]. Body mass index (BMI) was calculated as [weight/height^2^] and standardized BMI values were derived according to WHO standards (http://www.who.int/childgrowth/standards/en/).

### Statistical Analysis

Statistical analyses were carried out with PASW Statistics 18. Simple linear regression analysis was used to define independent variables associated with S-25-OHD concentration. After stratification into age groups we investigated seasonal variation in S-25-OHD concentration with a generalized linear model (ANCOVA) adjusting for calendar year. We also analysed age-related differences with ANCOVA using year and season as covariates. The four seasons were defined in the following way: from March to May as “spring”, from June to August as “summer”, from September to November as “autumn” and from December to February as “winter”. S-25-OHD, P-PTH and P-Ca were not normally distributed, and nonparametric Spearmańs rho was used to test correlations between variables. PTH was normally distributed within the reference range (8–73 ng/L).

In order to exclude patients with high S-25-OHD due to renal insufficiency and hydroxylation defect we excluded patients (N = 16) with plasma creatinine concentration exceeding the upper age-specific reference limit by >20% when assessing vitamin D status and seasonal variation. Subjects in the final cohort (N = 1335) were divided into five groups according to age: 0.0–2.0 years (N = 129), 2.1–6.0 years (N = 185), 6.1–10.0 years (N = 229), 10.1–15.0 years (N = 473) and 15.1–18.0 years (N = 319) (Table 1). This grouping was regarded appropriate as the national guidelines recommend daily vitamin D supplementation to those aged 0–2 years. After age 6 years children start school and age 10–15 years coincides with fastest pubertal development.

**Table 1:** Frequency of laboratory measurements in the study population.

		N (total 1335)	
Gender	boys	674	50.5%
	girls	661	49.5%
Age group (years)	0–2.0	129	9.7%
	2.1–6.0	185	13.9%
	6.1–10.0	229	17.2%
	10.1–15.0	473	35.4%
	15.1–18.0	319	23.9%
Season*	winter	362	27.1%
	spring	343	25.7%
	summer	241	18.1%
	autumn	389	29.1%
Year	2007	452	33.9%
	2008	300	22.5%
	2009	256	19.2%
	2010	327	24.5%

* winter = December to February, spring = March to May, summer = June to August, autumn = September to November.

In post-hoc analysis differences in diagnosis of the underlying disease, age, ethnic background, anthropometry, use of vitamin D supplements and the season of blood sampling were studied between the HIGH and LOW groups. Chi Square test was used to assess the significance between bivariate or nominal variables, while independent T test was applied in case of continuous variables.

## Results

### S-25-OHD Concentration

Altogether 1335 patients were included in the study (50.5% boys). Their mean age was 10.6±5.2 years (range 0.2–18). The distributions of S-25-OH measurements according to age group, season, etc. are presented in Table 1.

**Figure 1:**
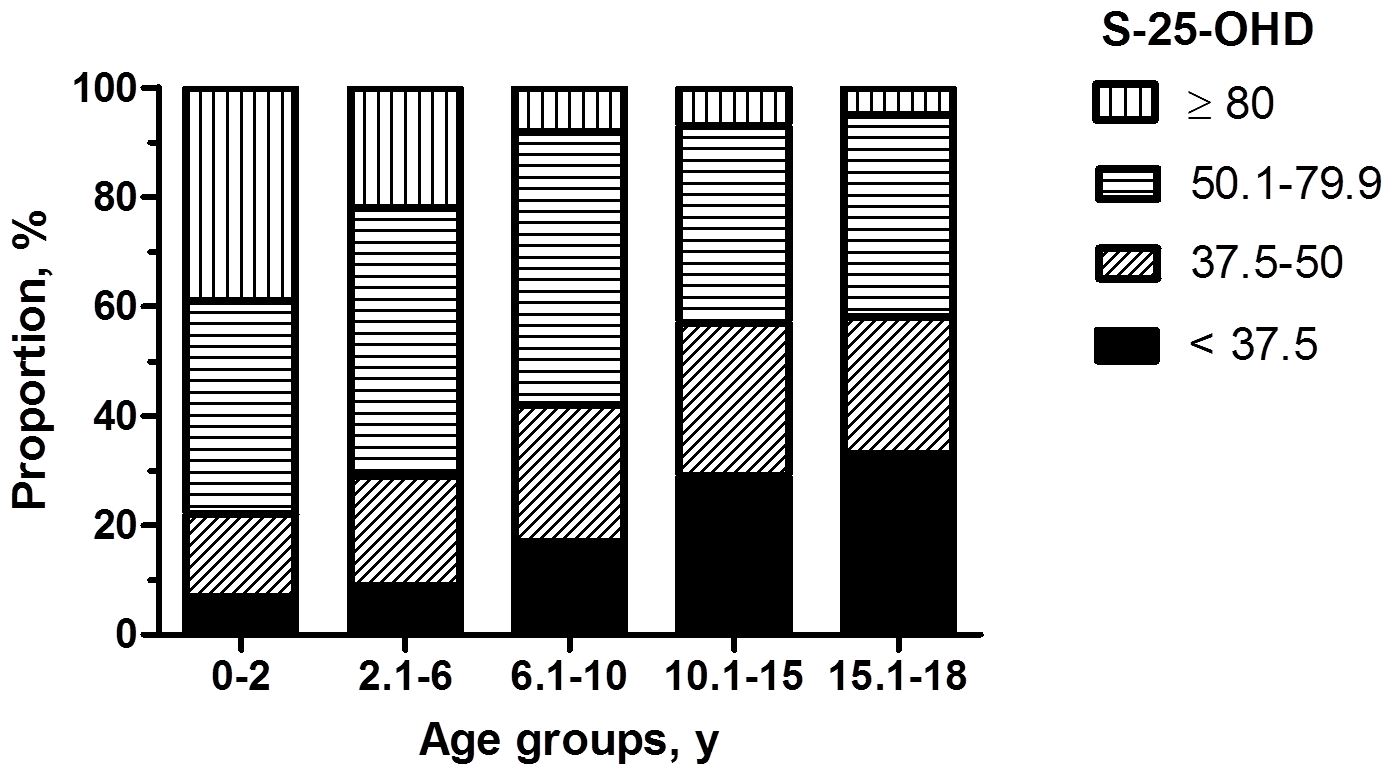
The prevalence of deficient, insufficient, sufficient, and optimal vitamin D status in each age group during 2007–2010.

Of the patients 47% had vitamin D deficiency or insufficiency. Altogether 23% of the subjects had S-25-OHD below 37.5 nmol/L and 24% between 37.5 to 50.0 nmol/L. In contrast, only 12% had a concentration above 80 nmol/L (Figure 1). Determinants of S-25-OHD concentration were tested with simple linear regression. Variables included age, year of measurement, season, and gender. Of these the most powerful predictor was age (standardized Beta −0.354, p<0.001), year of sampling (standardized Beta 0.142, p<0.001), and season (standardized Beta 0.122, p<0.001). Because gender was not a statistically significant predictor (p = 0.165) it was not regarded as a confounder in the analysis. Further analyses were performed by stratifying for age.

Mean S-25-OHD concentration differed significantly between age groups (Kruskal-Wallis; p<0.001): the highest concentrations were seen in the youngest and the lowest values in the oldest age groups. However, year and season remained confounding factors. Correspondingly, the prevalence of vitamin D deficiency was lowest (7%) in the 0–2 year olds and highest (33%) in the age group of 15.1–18.0 years (Table 2).

**Table 2:** Prevalence of vitamin D deficiency and insufficiency according to season*.

Vitamin D deficiency (S-25-OHD <37.5 nmol/L)							
		Season					
Age group (y)	N	Winter	Spring	Summer	Autumn	Total	P†
0–2.0	129	10%	9%	3%	6%	7%	Ns
2.1–6.0	185	8%	12%	10%	6%	9%	Ns
6.1–10.0	229	19%	22%	11%	12%	17%	Ns
10.1–15.0	473	37%	36%	13%	24%	29%	<0.001
15.1–18.0	319	42%	47%	11%	27%	33%	<0.001
Total	1335	29%	29%	11%	19%	23%	<0.001
**Vitamin D insufficiency (S-25-OHD 37.5–50 nmol/L)**							
		**Season**					
**Age group (y)**	**N**	**Winter**	**Spring**	**Summer**	**Autumn**	**Total**	**P** †
0–2.0	129	13%	14%	14%	18%	15%	ns
2.1–6.0	185	31%	16%	13%	15%	20%	ns
6.1–10.0	229	27%	33%	11%	22%	25%	ns
10.1–15.0	473	29%	31%	24%	28%	28%	ns
15.1–18.0	319	27%	19%	17%	32%	25%	ns
Total	1335	27%	25%	17%	25%	24%	0.044

† Pearson Chi-Square.

* winter = December to February, spring = March to May, summer = June to August, autumn = September to November.

Children and adolescents aged from 6 to 18 years showed a significant seasonal variation in S-25-OHD concentration (ANCOVA; p<0.001) after adjusting for calendar year, but this was not seen in younger age groups (Table 3). The highest mean concentrations of S-25-OHD were measured in the summer and these were on average 13 nmol/L higher than in the winter and spring. On the other hand children under 2 years of age and adolescents 15–18 years had significant variation between years, when using season as a covariate (ANCOVA; p = 0.001). A tendency towards higher S-25-OHD concentrations in 2010 than in other yeas was noted in all age groups. In the whole group prevalence of vitamin D deficiency and insufficiency varied across seasons (Chi Square; p = 0.001 and p = 0.044, respectively) (Table 3). Vitamin D deficiency was most commonly observed in two oldest age groups during winter and spring when 36% and 45% of adolescent were regarded as vitamin D deficient while least likely to suffer from deficiency was the youngest age group.

**Table 3:** Variation in mean serum 25-hydroxyvitamin D concentration (nmol/L) in each age group according to season*.

Age group (y)	N	Winter	Spring	Summer	Autumn	P‡
0–2.0	129	70	75	80	71	0.518
2.1–6.0	185	63	62	73	63	0.175
6.1–10.0	229	53	50	67	58	<0.001
10.1–15.0	473	45	46	57	52	<0.001
15.1–18.0	319	44	42	57	49	<0.001
Total	1335	51	51	63	55	<0.001

* winter = December to February, spring = March to May, summer = June to August, autumn = September to November.

‡ Analysis of covariance (ANCOVA) adjusted for calendar year.

### PTH

Age was the only significant determinant of PTH concentration in a simple linear regression when using age, year, season, and gender as independent variables. Thus, PTH concentration varied with age (ANOVA; p = 0.005) and showed increasing values with age (data not shown). Patients with PTH concentration above the reference range (>73 ng/L) (N = 108) were older than others (mean age 11.7±5 years vs. 10.3±5 years; T-test: p = 0.007).

**Figure 2:**
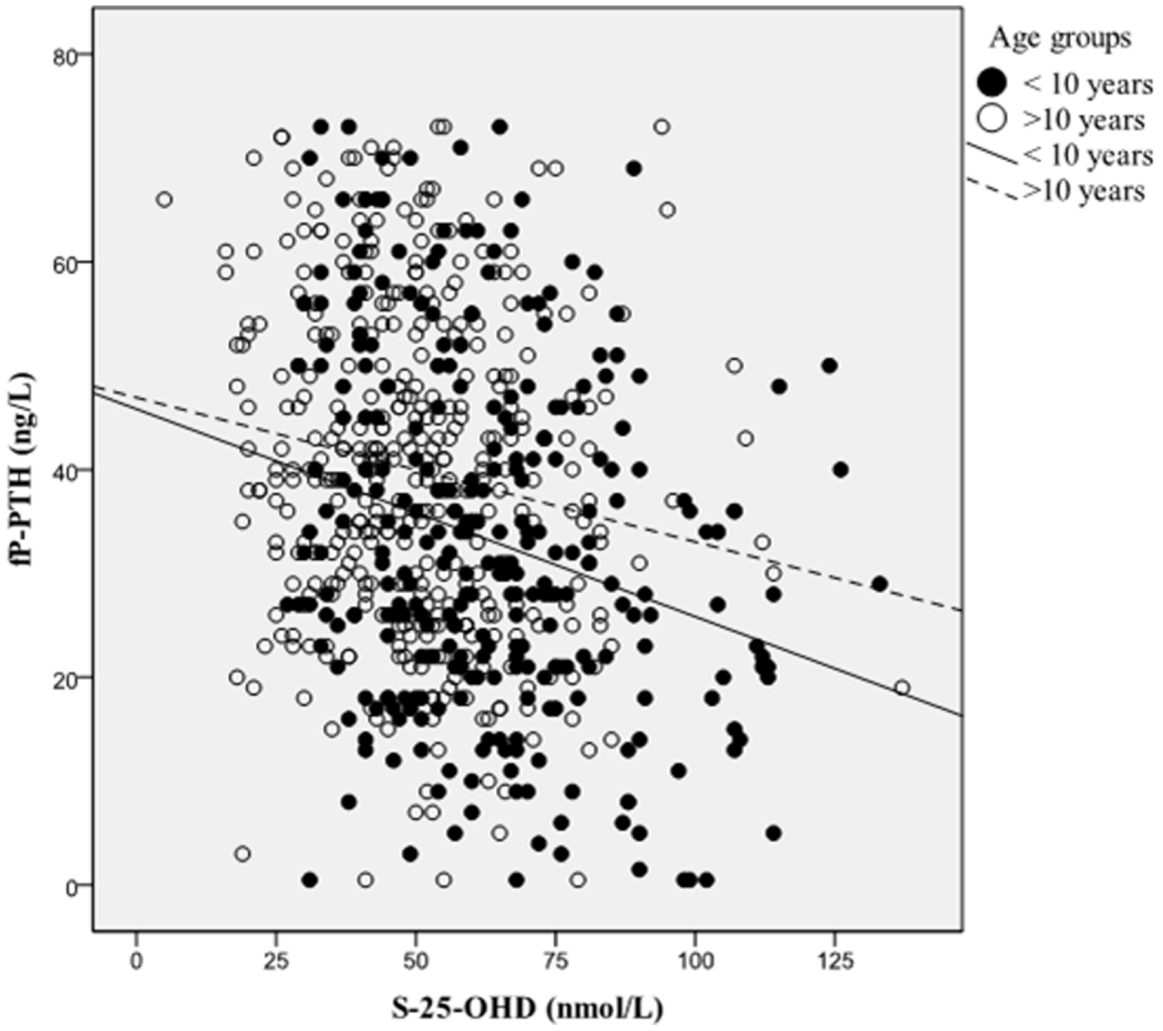
Correlation between fP-PTH (ng/L) and S-25-OHD (nmol/L) in children under and over 10 years. Cases with PTH >73 ng/L are excluded. Pearson correlation coefficients were −0.249 (p<0.001) and −0.163 (p = 0.001) for children under and over 10 years, respectively.

In the study population S-25-OHD correlated inversely with P-PTH (Spearman’s r = −0.250, p<0.001). The correlation remained the same when only those with PTH within the reference range (8–73 ng/L) were included (Pearson correlation −0.254, p<0.001). We observed that children under 10 years had stronger correlation between PTH and S-25-OHD concentrations than older children (Figure 2).

### Post-hoc Analysis

The groups of LOW and HIGH included altogether 100 children, whose characteristics are presented in Table 4. S-25-OH levels in the LOW group ranged from 5 nmol/L to 20 nmol/L and in the HIGH group between 103 nmol/L and 168 nmol/L. Sex distribution was similar in the groups (p = 0.69).

**Table 4:** Characteristics of the 100 patients in the LOW and HIGH vitamin D groups. The values are given as mean (SD).

	LOW	HIGH	P**
N	50	50	
S-25-OHD (nmol/L)	15.3 (4.1)	119 (16.9)	<0.001
Females (%)	46	56	0.689†
Non-Finnish ethnicity(%)	36.0	4.0	<0.001†
Age (years)	13.7 (3.5)	5.9 (5.5)	<0.001
Height (cm)	155.0 (23.0)	102.7 (35.1)	<0.001
Height Z-score	−0.4 (1.6)	−1.5 (1.8)	0.002
Weight (kg)	48.3 (24.0)	19.5 (15.5)	<0.001
Height-adjusted weight(%)	+4.7 (28.4)	−4.1 (14.0)	0.054
BMI (kg/m^2^)	19.2 (5.7)	16.0 (2.4)	<0.001
BMI Z-score	−0.42 (1.89)	−0.80 (1.55)	0.268
Use of vitamin D (%)	22.9	87.5	<0.001†

** Independent T-test.

† Pearson Chi- Square.

The subjects in the LOW group were significantly older, heavier, taller, and had higher BMI than those in the HIGH group (Table 4). Not only the absolute heights and weights were greater but also the age- and gender- adjusted heights (−0.4 SDS vs. −1.5 SDS, p = 0.002) and age-, gender- and height-adjusted weights (+4.7% vs. −4.1%, p = 0.054) were greater in the LOW group. On the other hand, no difference was observed in standardized BMI between groups. Number of subjects with standardized BMI<−2 or >2 indicating under- or overnutrition, respectively, were distributed similarly between the groups (p = 0.182).

Season affected the results: altogether 76% of the LOW values were obtained during winter or spring compared to 38% of the HIGH group, (p<0.001).

The use of vitamin D supplements (data available for 96 subjects) was fairly common as altogether 55% of the patients used vitamin D as a supplement; this did not differ between genders. Supplements were more common among the HIGH (88%) than LOW (23%) group (p<0.001) (Table 4). The use of supplements was especially common in the age group 0–2 years (N = 19) in which 89% used vitamin D supplements. In the oldest age group (12.1–18.0 years) 63% used vitamin D supplements.

Non-Finnish ethnic background was a risk factor for low 25-OHD concentration. In this post-hoc cohort 20 of the 100 subjects had non-Finnish background: non-Finnish ethnicity was more frequent in the LOW compared to the HIGH group (p<0.001)(Table 4). In addition, vitamin supplements were used by only five (25%) of those with non-Finnish background, compared to 59% of subjects with Finnish background.

**Figure 3:**
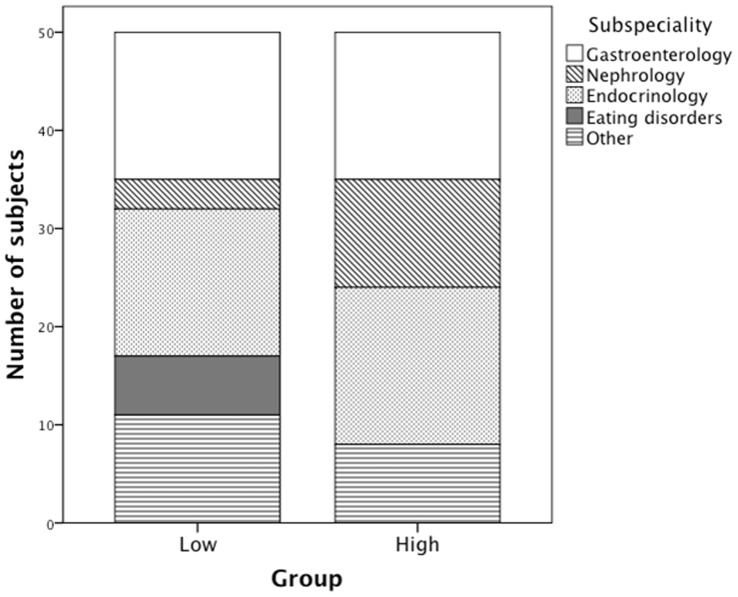
Underlying chronic illnesses in the LOW and HIGH vitamin D status groups.

Patients with extreme 25-OHD concentrations represented gastroenterology (30%), endocrinology (31%), nephrology (14%), eating disorders (6%) and other pediatric subspecialities (19%) (Figure 3). Patients from gastroenterology and endocrinology were equally represented in the LOW and HIGH groups. Notably 11 of 14 subjects with a renal disorder belonged to the HIGH group and six of them had increased P-Crea. Supranormal creatinine levels suggest that in these subjects high S-25-OHD values may partly reflect impaired renal vitamin D hydroxylation and in such a case suggest poor rather than good vitamin D status despite high S-25-OHD. Patients with eating disorders were prevalent in LOW group.

None of the patients had hypercalcemia; P-Ca and P-Pi levels were similar in the LOW and HIGH groups. PTH levels were significantly higher in the LOW than in the HIGH group, even when the subjects with increased S-Cr were omitted from the analysis (77.7 vs. 36.2, p = 0.002).

## Discussion

Our study involving more than 1300 children and adolescents with a chronic illness shows an alarmingly high prevalence of vitamin D deficiency among patients followed in a large pediatric centre. Age determined most of the variation in serum 25-OHD concentration: the prevalence of vitamin D deficiency was highest among adolescents and lowest in infants. In addition to older age, lack of vitamin D supplementation and non-Finnish ethnic background were risk factors for poor vitamin D status. We observed significant seasonal variation in S-25-OHD concentration, still 28% of the outpatients remained vitamin D deficient or insufficient during the summer.

Almost 80% of infants had S-25-OHD concentration above 50 nmol/L. Our finding is consistent with a prospective study in which most infants aged 14 months had sufficient vitamin D status [18]. This implies that during the first two years of life children usually receive adequate amount of vitamin D from diet and supplements. Families of young children are compliant with vitamin D supplementation, but the use of supplementation decreases while children grow older [19]. Despite chronic illnesses, the children under 6 years of age still had a mean concentration of S-25-OHD above 60 nmol/L and the prevalence of vitamin D deficiency ( =  S-25-OHD below 37.5 nmol/L) was only approximately 10%. The post-hoc analysis reinforced that vitamin D status in infants is better than in older children and adolescents.

Interestingly, no seasonal variation in S-25-OHD concentration in children under 6 years was noted, implying that compared to dietary supply the effect of sunlight exposure is very limited in younger Finnish children with a chronic illness. Vitamin D insufficiency has been under discussion in Finland during the last few years, and this may have improved parents’ awareness of the importance of vitamin D supplementation. Furthermore, liquid dairy products and spreads are currently fortified with vitamin D in Finland [20]. On 2010 the National Nutrition Council recommended an increase to the fortification level in liquid dairy products from 0.5 µg to 1 µg per 100 ml and in spreads from 10 µg to 20 µg per 100 g which were rapidly adopted by many dairy companies. These could explain the slight increase in S-25-OHD concentrations in 2010 in all age groups.

In older age groups the mean S-25-OHD was consistently below 50 nmol/L and the prevalence of vitamin D deficiency was almost four times higher than in younger age groups. Similar observations have been reported in healthy children [21], [22], but to our knowledge not in chronically ill patients. The prevalence of deficiency is in accordance with our finding in apparently healthy children and adolescents, of whom 39% suffered from deficiency during winter and spring [13]. Puberty is a critical period in bone development [23], [24] and vitamin D deficiency or even milder insufficiency may lead to increased risk of fractures and osteoporosis during childhood and adolescence [11], [24] but also later in adulthood. Some have described a frequent vitamin D insufficiency in pediatric outpatients [25], [26], but these have included rather small number of subjects. A report from NHANES demonstrated that vitamin D deficiency was associated with several cardiovascular risk factors in a pediatric cohort [22]. It is known that chronic illness in children may associate with delayed or impaired growth and pubertal development, but other, secondary health concerns such as metabolic syndrome [27] still remain inadequately characterized. Thus, despite careful monitoring of the disease, more attention should be paid on nutritional status which has a vast effect on well-being. Adolescents are likely to require higher intake of vitamin D to ensure sufficient vitamin D status. Although pediatric recommendations for children with chronic illness regarding the optimal S-25-OHD level are lacking patients with chronic illness may benefit from S-25-OHD concentrations above 80 nmol/L, as recommended by the Endocrine Society expert panel to attain multiple benefits [10].

Children and adolescents aged from 6 to 18 years showed significant seasonal variation in S-25-OHD concentration. Summer values were on average 13 nmol/L higher compared to winter values. Seasonal variation in vitamin D status has been reported in healthy children [28], [29]. Nevertheless, more than every fourth patient had subnormal vitamin D status during the summer months: 11% had vitamin D deficiency and 17% had vitamin D insufficiency. Helsinki is located at 60^○^N and therefore sun exposure from October to April is minimal and during this period vitamin D production in the skin is limited [30]. Children with chronic illness are less likely to spend time outside than healthy children and their sunlight exposure may be limited even in the summer.

Some studies have described optimal vitamin D status by maximal suppression on PTH [31], but PTH may not plateau at all [32]. We found an inverse correlation between PTH and S-25-OHD, but no plateau. The correlation was stronger in younger subjects than in adolescents older than 10 years of age. This suggests that during rapid pubertal growth PTH secretion is regulated by multiple factors besides S-25-OHD as described earlier [3]. Taken together this and the intermittent nature of PTH, it seems that PTH is not an outcome to base the definition of optimal vitamin D status in pediatric population.

Special concern should be paid to patients with non-Finnish ethnic background: in the subgroup analysis they were more likely to have extremely low 25-OHD concentrations and less likely to use of vitamin D supplements than native Finns. People with non-Finnish background usually have darker skin pigmentation and they require longer sunlight exposure for vitamin D synthesis [34]. Furthermore, the intake of fish, milk and milk products is very low in immigrant populations around Europe [35], thus the current fortification policy, involving mainly dairy products, may not be effective to them. Since the overall proportion of children and adolescents with non-Finnish background in the hospital area is very low, ethnic factors do not explain the overall poor vitamin D status in our study.

According to guidelines of the Finnish National Nutrition Council (2011), which were revised after our data collection period, children and adolescent under 18 years of age should receive daily supplementation of vitamin D (7.5–10 µg) throughout the year regardless of diet. Based on our findings these changes are timely: the use of vitamin D supplements prevents deficiency. It is surprising that the 50 highest 25-OHD concentrations measured in a large university hospital out-patient clinic over a 4-year-period, ranged only from 103 to 168 nmol/L This suggests that overall there is a great need to change the pediatric practices when using vitamin D supplements in patients with an underlying chronic illness. The levels are far from toxic and if only 12% of the measured values exceed 80 nmol/L it is clear that the vast majority of children with a chronic illness have suboptimal vitamin D status. All reports of vitamin D toxicity showing evidence of hypercalcemia involve serum 25-OHD concentrations significantly above 200 nmol/L and require daily intake of ≥1000 µg (40 000 IU) [7]. Thus, regular and more liberal use of vitamin D supplements within upper level of intake [9] should be encourages in pediatric patients. Furthermore, the current recommendations are intended only for healthy individuals. In the case of chronically ill children vitamin D supplementation should be considered individually and S-25-OHD concentration should be measured at least when diagnosis of chronic illness is made and during exacerbations.

Post-hoc analysis showed that patients with eating disorders are more likely to have low 25-OHD concentration while high values are common in renal disease. Previously chronic illnesses, such as inflammatory bowel disease [36], type 1 diabetes [37], juvenile idiopathic arthritis [38] and solid organ [39] and bone marrow transplantation [40] in children are associated with vitamin D deficiency. On the other hand, vitamin D deficiency or insufficiency may be a consequence of a chronic illness that affects vitamin D metabolism or absorption, such as biliary atresia and autoimmune hepatitis [41], chronic kidney disease [42], inflammatory bowel disease [36], or cancer [43]. Due to cross-sectional study design, we were not able to evaluate causality or the consequences of our findings.

Our study has several limitations. The group of chronically ill children in this study does not include all chronically ill patients visiting the Children’s Hospital, Helsinki. This register-based study included all pediatric outpatients who had S-25-OHD measured between 2007 and 2010. Sampling was based on the judgment of individual clinicians. Due to high prevalence of vitamin D deficiency in Finland assessment of vitamin D status has become an established part of the clinical follow-up and therefore sampling bias is unlikely. Based on our large sample size, the study represents and evaluates reliably the vitamin D status in chronically ill outpatient children in Finland. The retrospective hospital register-based approach prevented us from collecting all relevant information, including dosing of and compliance with vitamin D supplements, dietary intake of vitamin D and confounding factors such as inflammatory status. The post-hoc analysis was limited and due to the large number of various underlying chronic illnesses in the study population and the relatively small sample size in post-hoc analysis we were not able to demonstrate significant differences in vitamin D status between the diagnostic groups.

We conclude that vitamin D deficiency is alarmingly prevalent in pediatric patients, especially in adolescents, with a chronic illness. Despite marked seasonal variation, S-25-OHD concentrations are low throughout the year and even in summer 27% of pediatric outpatients remain vitamin D deficient or insufficient. Predisposing factors for vitamin D deficiency include older age, non-Finnish background, lack of vitamin D supplementation and winter. Clinicians responsible for the follow-up of pediatric patients should be aware of the problem and actively monitor vitamin D status of their patients to ensure vitamin D sufficiency.
